# The Direct/Indirect Association of ADHD/ODD Symptoms with Self-esteem, Self-perception, and Depression in Early Adolescents

**DOI:** 10.3389/fpsyt.2017.00137

**Published:** 2017-07-31

**Authors:** Yosuke Kita, Yuki Inoue

**Affiliations:** ^1^Department of Developmental Disorders, National Institute of Mental Health, National Center of Neurology and Psychiatry (NCNP), Tokyo, Japan; ^2^Department of Child Psychiatry, Shimada Ryoiku Center Hachioji, Tokyo, Japan; ^3^Department of Child Psychiatry, Yokohama City Southern Area Habilitation Center for Children, Yokohama, Japan

**Keywords:** attention-deficit hyperactivity disorder, oppositional defiant disorder, depression, self-esteem, structural equation modeling

## Abstract

The present study aimed to reveal the influences of attention-deficit hyperactivity disorder (ADHD) and oppositional defiant disorder (ODD) symptoms on self-esteem and self-perception during early adolescence and to clarify the spillover effect of self-esteem on depressive symptoms. ADHD symptoms in 564 early adolescents were evaluated *via* teacher-rating scales. Self-esteem and depressive symptoms were assessed *via* self-reported scales. We analyzed the relationships among these symptoms using structural equation modeling. Severe inattentive symptoms decreased self-esteem and hyperactive–impulsive symptoms affected self-perception for non-academic domains. Although these ADHD symptoms did not directly affect depressive symptoms, low self-esteem led to severe depression. ODD symptoms had a direct impact on depression without the mediating effects of self-esteem. These results indicated that inattentive symptoms had a negative impact on self-esteem and an indirect negative effect on depressive symptoms in adolescents, even if ADHD symptoms were subthreshold. Severe ODD symptoms can be directly associated with depressive symptoms during early adolescence.

## Introduction

Attention-deficit hyperactivity disorder (ADHD) is a neurodevelopmental condition with an estimated prevalence of 5% in children worldwide (1). ADHD is characterized by a developmentally inappropriate level of inattention and hyperactivity/impulsivity. It persists from childhood to adulthood, interfering with functioning in several domains (2). Academic and educational outcomes are significantly lower in children with ADHD. For example, ADHD is associated with poor grades, increased rates of detention and expulsion, and low rates of high school graduation (3). Children with ADHD are also likely to experience a greater number of negative social events, such as being rejected by peers, having poor social skills (4), and having frequent negative interactions with their mothers (5).

These negative experiences can have a large impact on self-esteem in children with ADHD. According to the multidimensional model of self-esteem (6), children who frequently experience failure are at risk of developing a lower sense of self-competence. Conversely, children who often experience success may develop an enhanced sense of self-efficacy. Over the past three decades, a number of studies focused on self-esteem in children with ADHD [for review, see Ref. (7, 8)] have led to the recognition of self-esteem as an important topic affecting individuals with ADHD. According to one such review (8), more than half of previous studies found that children with ADHD had lower self-esteem compared with healthy controls. However, some children with ADHD rate their quality of life as being less negative compared with evaluations made by their parents (7). Children with ADHD also tend to overestimate their own competence [for review, see Ref. (9, 10)]. Thus, it is unclear whether children with ADHD symptoms have low self-esteem during childhood owing to negative life experiences.

Previous studies have consistently reported low self-esteem in adults with ADHD [for review, see Ref. (11)]. Additionally, studies exploring gender differences in self-esteem found a decrease in this population regardless of gender (12–14). Two studies (15, 16) exploring gender differences pointed out that self-esteem in adults with ADHD is lower for those with combined type vs. inattentive type (15). Based on these findings regarding childhood and adulthood, we speculated that people with ADHD symptoms follow a developmental course in which their self-esteem gradually decreases until adulthood, even if they have high self-esteem during childhood.

Relatively, few studies have focused on adolescent ADHD. While previous studies have discussed the relationships between ADHD symptoms and self-esteem in both children and adults, the relationship between these two developmental stages is unclear. One study found a significant relationship between parent-rated and self-reported ADHD symptoms in adolescents aged 13 (17). Scholtens et al. (18) conducted a community sample study and reported that ADHD symptoms had a negative effect on academic progress and academic self-perception. Additionally, Glass et al. (19) examined the relationship between self-esteem and conduct problems in adolescents with ADHD and reported that self-esteem was significantly lower in adolescents with ADHD and conduct problems but not in adolescents with ADHD only. Based on these findings, we hypothesized that self-esteem in people with ADHD symptoms decreases with age from childhood to adulthood and that adolescence is a transitional stage in which ADHD symptoms affect both self-esteem and internal problems such as depressive symptoms. This is likely related to the gradual increase in the total amount of negative experiences related to ADHD symptoms along the lifespan of an individual. We sought to fill in the gaps between childhood and adulthood, focusing on the relationship among ADHD symptoms, self-esteem, and internal behavioral problems.

The first aim of the current study was to examine the influence of ADHD symptoms on self-esteem in early adolescence, with a focus on school-based samples. Because clinical adolescent participants with ADHD often have very complicated mental and/or behavioral problems, evaluations of ADHD symptoms might be distorted by comorbid disorders in such samples. Moreover, recent studies suggested that ADHD symptoms are considered as a continuum rather than a dichotomous condition (20, 21), which encouraged us to focus on typically developing adolescents who have subthreshold ADHD symptoms. Thus, we performed a school-based study to investigate the relationships between ADHD symptoms and self-esteem in adolescents.

The second aim of the current study was to clarify the relationships between self-esteem and other clinical symptoms, such as behavioral and depressive symptoms, which are frequently comorbid with ADHD in adolescents. While ADHD symptoms are thought to affect self-esteem, behavioral problems such as oppositional defiant disorder (ODD) are also known to be contributing factors. Previous studies have indicated that co-occurring aggressive behavior is related to enhanced self-estimations [for review, see Ref. (9)]. Moreover, self-esteem usually impacts depressive symptoms, such that lower self-esteem can easily lead to severe depressive symptoms in children (22). This tendency appears to be more pronounced in adolescents compared with children, which is expected based on the typical developmental trajectory (23).

**Figure 1:**
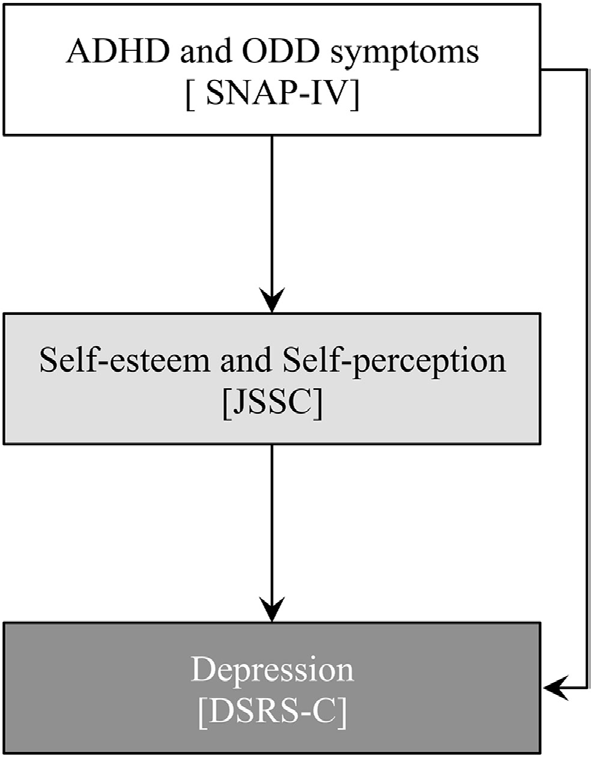
Hypothetical model of relationships between attention-deficit hyperactivity disorder (ADHD) and oppositional defiant disorder (ODD) symptoms, self-esteem, self-perception, and depression.

Given the aims stated previously, we investigated the relationships among ADHD symptoms, ODD symptoms, self-esteem, and depressive symptoms in adolescents based on our hypothetical model (Figure 1). As illustrated in Figure 1, we expected that ADHD and ODD symptoms affect self-esteem and self-perception, which have been shown in previous studies about children and adults with ADHD (7, 11). Self-esteem and self-perception would have influences on depressive symptoms in adolescents (20, 21). Moreover, the depressive symptoms might be affected by ADHD and ODD symptoms because the high rates of the comorbidity of ADHD/ODD and depression have been also reported in clinical samples (24, 25). We had these hypothetical associations and conducted our analysis *via* structural equation modeling (SEM).

## Materials and Methods

### Participants and Procedure

Participants comprised 564 children, ranging in age from 12 to 15 years, from two local public middle schools. Prior to engagement in the study, we provided sufficient explanations about the details of the study to the school principals, children, and their parents. We obtained school approval and written informed consent from all schools and parents, respectively, prior to study participation. To limit our participant sample to typically developing children without severe neurological or psychiatric disorders, we did not include children engaged in special education. Some children made mistakes in their responses, resulting in a valid response rate of 66.4%. The rate seemed to be relatively low because we adopted listwise deletions of cases for the statistical analyses (i.e., we excluded children who have at least one missing value in all questionnaires). Thus, the final sample comprised 374 children (194 boys and 180 girls). The average age of the participants was 13.69 years (SD = 0.89) (Table 1), and all participants were native-born Japanese children. The research protocol was approved by the ethics committee at the Shimada Ryoiku Center Hachioji (Tokyo, Japan; approval number 201503).

**Table 1:** Number of participants (upper lines) and mean ages with standard deviations (M ± SD; lower lines).

	Boy	Girl	Sum
First grade (12–13 years)	65	70	135
	12.75 ± 0.29	12.67 ± 0.28	12.71 ± 0.29
Second grade (13–14 years)	54	52	106
	13.67 ± 0.27	13.68 ± 0.3	13.67 ± 0.29
Third grade (14–15 years)	75	58	133
	14.66 ± 0.27	14.73 ± 0.27	14.69 ± 0.27
Sum	194	180	374
	13.75 ± 0.86	13.62 ± 0.91	13.69 ± 0.89

### Measures

#### ADHD and ODD Symptoms

We used a Japanese version of the Swanson, Nolan, and Pelham Rating Scale-IV (SNAP-IV) Teacher form (26) to assess symptoms of ADHD and ODD. The original version of the SNAP-IV was used as a primary outcome measure in the Multimodal Treatment of Attention Deficit Hyperactivity Disorder study (27, 28), and has been translated into languages other than English with high reliability and validity (26, 29). The SNAP-IV is a 26-item questionnaire that comprises three factors: Inattention (9 items), Hyperactivity–Impulsivity (9 items), and ODD (8 items). Each item is measured on a four-point Likert scale ranging from 0 (“not at all”) to 3 (“very much”), and each factor score is calculated by taking the mean of the items. Higher scores on the factors reflect more severe symptoms, and we set the cutoff values for Inattention and Hyperactivity–Impulsivity as 2.56 and 1.78, respectively. Classroom teachers, who spent over 20 h/week with the children, evaluated the participant symptoms. The SNAP-IV was administrated to them in their school as a paper-and-pencil questioner.

#### Self-esteem and Self-perception

We measured self-esteem and self-perception *via* the Japanese version of the Scale of Self-Cognition [JSSC (30)]. The JSSC is a 26-item self-reported questionnaire for children ranging in age from 8 to 15 years, and was translated from the original “Self-Perception Profile for Children” scale (6, 31). The JSSC has two main factors: Global Self-Worth (i.e., self-esteem) and domain-specific judgments (i.e., self-perception). The former factor is assessed *via* six items, and the latter factor is divided into five specific domains: Scholastic Competence (five items), Athletic Competence (four items), Behavioral Conduct (three items), Social Acceptance (three items), and Physical Appearance (five items). Children were asked to respond to each item using a four-point Likert scale ranging from 1 (“not at all”) to 4 (“very much”) when they were in school (i.e., a paper-and-pencil questioner). Scores for each of six subscales (i.e., one for self-esteem and five for self-perception) were calculated by taking the mean of the items. Higher scores on the subscales reflected higher levels of self-esteem or self-perception.

#### Depression

We assessed depression in children *via* the Japanese version of the Depression Self-Rating Scale for Children (DSRS-C (32)). The DSRS-C has high validity and reliability (33, 34) and is suitable for children because it consists of just 18 easy items. Children use a three-point Likert scale ranging from 0 (“not at all”) to 2 (“most of the time”) to respond to each item when they were in school (i.e., a paper-and-pencil questioner). Depression score is calculated by summing the item scores. Higher scores indicate more severe depressive symptoms, and we set a cutoff score of 16 for identifying depressed children (32).

### Data Analysis

We generated descriptive statistics for each measurement. We also calculated the percentages of children with scores above the cutoff values for Inattention and Hyperactivity–Impulsivity in the SNAP-IV, and Depression in the DSRS-C. We calculated Pearson’s correlation coefficients to examine the relationships among all valuables. We examined gender differences for three factors in the SNAP-IV, Depression scores, and Global Self-Worth using one-way *t*-tests. We tested self-perception *via* a two-way repeated measures ANOVA [gender (2) × domains (5)] followed by *post hoc* analysis with the Bonferroni adjustment. We used SEM to study our hypothesis regarding the relationship between ADHD and ODD symptoms, depression, self-perception, and self-esteem. We examined the suitability of our hypothetical model using maximum likelihood estimation techniques. We used multiple fit indices, such as the ratio between the chi square statistic and the Degrees of Freedom (χ^2^/*df*), the Goodness-of-Fit Index (GFI), the Adjusted Goodness-of-Fit Index (AGFI), the Comparative Fit Index (CFI), and the Root Mean Square Error of Approximation (RMSEA) to evaluate model fit. We set the statistical criteria for a good fit between the model and data as follows: χ^2^/*df* < 5 (35), GFI > 0.95, AGFI > 0.95, CFI > 0.95, and RMSEA < 0.05 (35, 36). We examined each path in the model using Wald tests with significance set at *p* = 0.05. Statistical analyses were conducted using IBM SPSS Statistics 19 (SPSS Japan Inc., Tokyo, Japan) and IBM SPSS Amos 19 (SPSS Japan Inc., Tokyo, Japan).

## Results

### Descriptive Statistics and Correlations

The average scores for ADHD and ODD symptoms were close to 0, as follows: Inattention = 0.23 ± 0.42 (mean ± SD), Hyperactivity–Impulsivity = 0.06 ± 0.22, and ODD = 0.08 ± 0.25. The mean score for Depression was also lower than the cutoff value: 9.94 ± 5.26. The results for self-esteem and self-perception were as follows: Global Self-Worth = 2.58 ± 0.65, Scholastic Competence = 2.42 ± 0.65, Athletic Competence = 2.12 ± 0.80, Behavioral Conduct = 2.50 ± 0.60, Social Acceptance = 3.20 ± 0.66, and Physical Appearance = 2.29 ± 0.71. While all children had scores below the cutoff values for Inattention (i.e., 100.0% of the total sample), one child had scores above the cutoff value for Hyperactivity–Impulsivity (i.e., 0.3% of the total sample). 52 children (13.9% of the total sample; 18 boys and 34 girls) were identified as depressed because they obtained scores above the cutoff value for Depression.

Table 2 presents the correlations among all variables. ADHD and ODD symptoms were positively correlated with one another (*r*s > 0.59, *p*s < 0.001). These symptoms were also negatively associated with self-perception in terms of Scholastic Competence (vs. Inattention *r* = −0.26, *p* < 0.001; vs. Hyperactivity-Impulsivity, *r* = −0.18, *p* < 0.001; vs. ODD, *r* = −0.10, *p* < 0.05) and Behavioral Conduct (vs. Inattention *r* = −0.25, *p* < 0.001; vs. Hyperactivity-Impulsivity, *r* = −0.26, *p* < 0.001; vs. ODD, *r* = −0.22, *p* < 0.001). While ODD symptoms were positively associated with Depression (*r* = 0.21, *p* < 0.001), this was not the case for both Inattention and Hyperactivity–Impulsivity (*p*s > 0.05). We found significant positive correlations between all associations with respect to the six factors in the JSSC (*r*s > 0.14, *p*s < 0.01) except that between Behavioral Conduct and Social Acceptance (*r*s = 0.01, *p* > 0.05). Depression was negatively associated with all factors in terms of self-esteem and self-perception (*r*s < −0.25, *p*s < 0.001).

**Table 2:** Correlation coefficients among the variables.

	Age	Inattention	Hyperactivity-Impulsivity	ODD	Scholastic Competence	Athletic Competence	Behavioral Conduct	Social Acceptance	Physical Appearance	Global Self-worth	Depression
Age	–	−0.03	−0.08	−0.11*	0.03	0.06	0.26***	−0.06	−0.08	−0.05	0.03
Inattention		–	0.66***	0.59***	−0.26***	−0.04	−0.25***	0.10*	−0.04	−0.07	0.07
Hyperactivity-Impulsivity			–	0.62***	−0.18***	0.07	−0.26***	0.12*	0.02	0.04	0.05
ODD				–	−0.10*	0.04	−0.22***	0.09	−0.05	−0.04	0.21***
Scholastic Competence					–	0.33***	0.38***	0.14**	0.43***	0.43***	−0.33***
Athletic Competence						–	0.21***	0.47***	0.43***	0.45***	−0.37***
Behavioral Conduct							–	0.01	0.25***	0.35***	−0.25***
Social Acceptance								–	0.20***	0.28***	−0.38***
Physical Appearance									–	0.60***	−0.40***
Global Self-worth										–	−0.54***

*p < 0.05.

**p < 0.01.

***p < 0.001.

### Gender Differences

**Figure 2:**
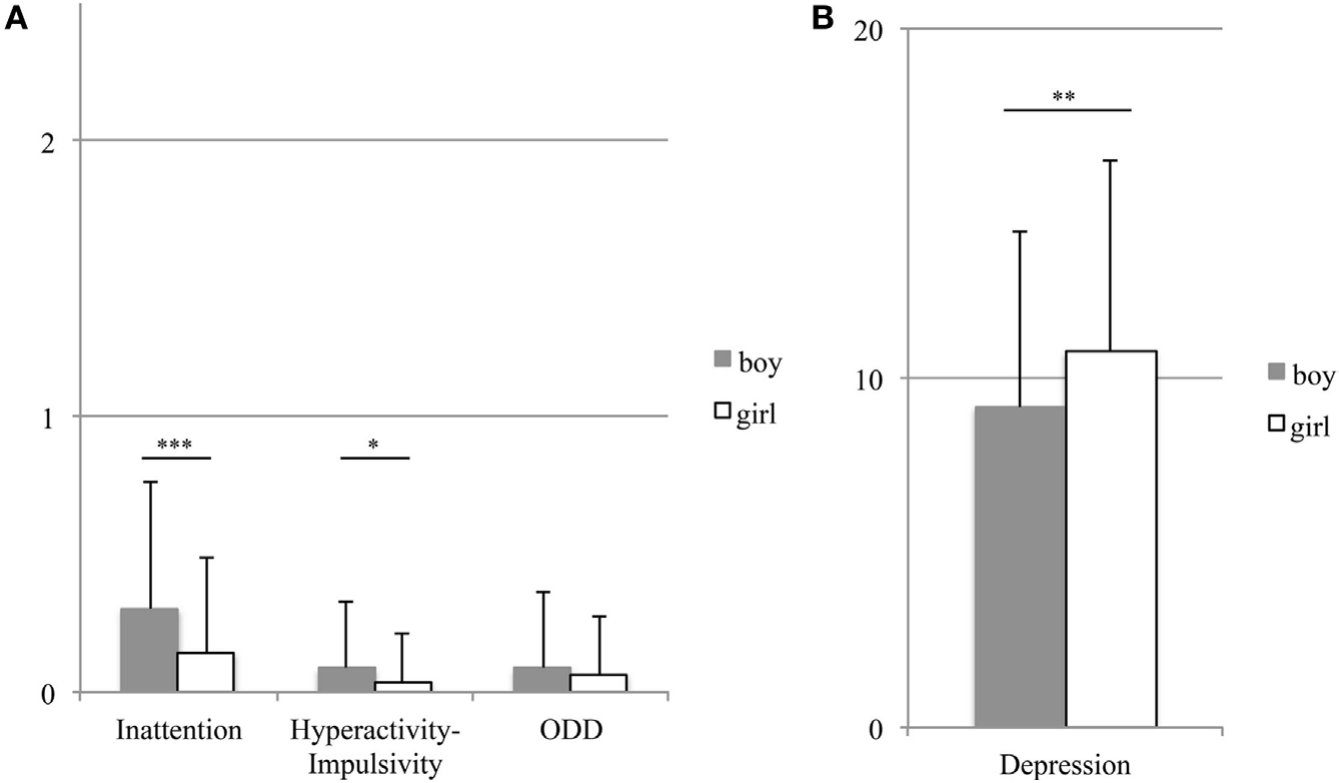
Gender differences in attention-deficit hyperactivity disorder (ADHD) symptoms **(A)** and depression **(B)**. The bars and error bars show the means and standard deviations, respectively. ***p < 0.001, **p < 0.01, and *p < 0.05.

**Figure 3:**
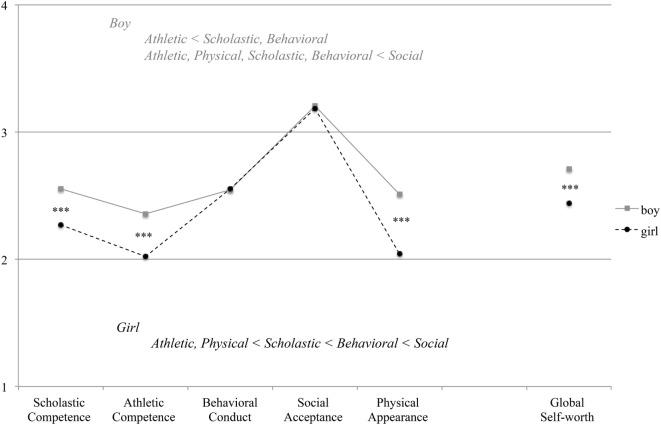
Gender differences in self-esteem and self-perception for five domains. ***p < 0.001.

Compared with girls, boys obtained higher scores for Inattention [*t*(372) = 3.73, *p* < 0.001] and Hyperactivity–Impulsivity [*t*(372) = 2.26, *p* = 0.024]. Gender differences were not significant for ODD score [*t*(372) = 0.87, *p* = 0.383] (Figure 2A). However, girls obtained a higher Depression score [*t*(372) = 2.670, *p* = 0.008] (Figure 2B) and a lower score for Global Self-Worth [*t*(372) = 4.11, *p* < 0.001]. Figure 3 shows comparisons of self-perception scores between the genders. A two-way repeated measures ANOVA revealed a significant interaction between gender and domain [*F*(4, 1,488) = 12.111, *p* < 0.001]. *Post hoc* analyses indicated that boys scored higher than girls with respect to Scholastic Competence, Athletic Competence, and Physical Appearance (*p*s < 0.001). For boys, the highest score was for Social Acceptance and the lowest score was for Athletic Competence. Girls received scores in the following order, ranked from highest to lowest: Social Acceptance, Behavioral Conduct, Scholastic Competence, Physical Appearance, and Athletic Competence (*p*s < 05).

### Relationships among ADHD and ODD Symptoms, Depression, Self-perception, and Self-esteem

**Figure 4:**
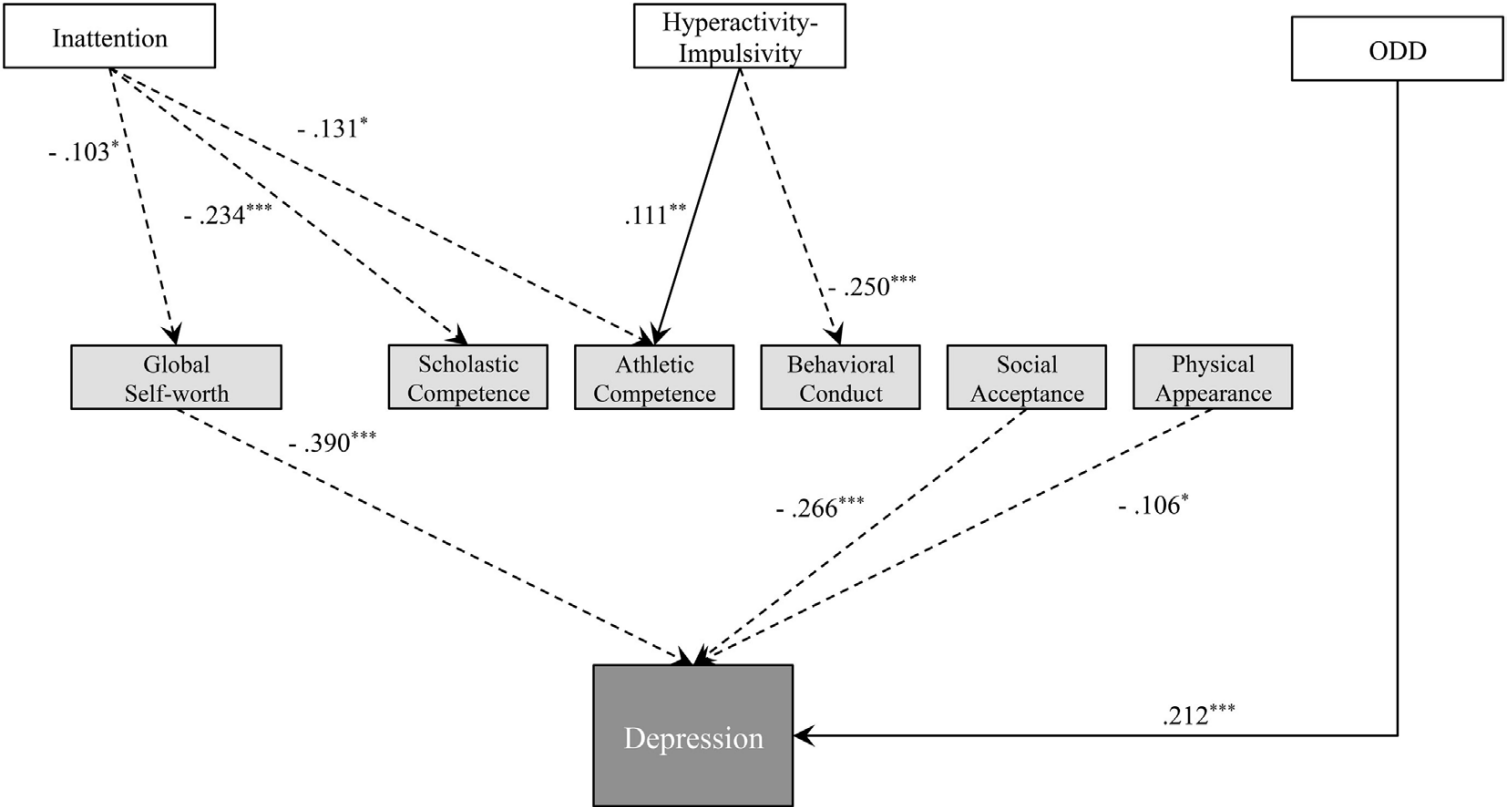
Final model of relationships between attention-deficit hyperactivity disorder (ADHD) and oppositional defiant disorder (ODD) symptoms, self-esteem, self-perception, and depression with standardized path coefficients. The solid and dashed arrows show the positive and negative effects, respectively. ***p < 0.001, **p < 0.01, and *p < 0.05.

As a first test step, we used the SEM to evaluate our hypothetical model (Figure 1), which set all paths between the observed variables. This first test step produced several inadequate values of fit indices, such as χ^2^/*df* = 41.72 and GFI = 0.62. Thus, we modified the first model by removing paths that were insignificant. Moreover, we added the residual error covariance between factors associated with ADHD and ODD symptoms, and domains in self-perception, based on modification indices. We repeated these modification procedures to reach the final modified model, shown in Figure 4. The SEM produced fit indices that indicated a good fit between the final model and data [χ^2^ (18) = 31.37, *p* = 0.03; χ^2^/*df* = 1.743; GFI = 0.984; AGFI = 0.952; CFI = 0.989; RMSEA = 0.048].

All paths in the final model were significant according to the Wald test (*p*s < 0.05) (Figure 4). Inattention was associated with self-esteem and self-perception. Specifically, severe inattentive symptoms decreased Global Self-worth (path coefficient = −0.103), Scholastic Competence (path coefficient = −0.234), and Athletic Competence (path coefficient = −0.131). High levels of Hyperactivity–Impulsivity also decreased self-perception with respect to Behavioral Conduct (path coefficient = −0.250) but increased Athletic Competence (path coefficient = 0.111). We did not find any direct effects of ADHD symptoms on depression, although severe ODD symptoms were associated with severe depression (path coefficient = −0.212). Greater Global Self-Worth was strongly associated with decreased depression (path coefficient = −0.390). Moreover, greater self-perception regarding Social Acceptance and Physical Appearance, both of which were not influenced by ADHD symptoms, was associated with decreased depression (path coefficient = −0.266 and −0.106, respectively). However, Scholastic Competence, Athletic Competence, and Behavioral Conduct, which were self-perception domains influenced by ADHD symptoms, were not significantly associated with depression.

## Discussion

In the present study, we found that inattentive symptoms were associated with decreased Global Self-worth, Scholastic Competence, and Athletic Competence, while hyperactive–impulsive symptoms were associated with self-perception regarding Behavioral Conduct. Moreover, low self-esteem (i.e., Global Self-worth) and self-perception (Social Acceptance and Physical Appearance) were strongly associated with severe depression. Although ADHD symptoms were not directly associated with depressive symptoms, this was the case for ODD symptoms in early adolescents.

The present data indicate that ADHD symptoms in early adolescents are associated with self-esteem and several domains of self-perception. This is consistent with a previous survey conducted with a community sample (18). The authors found that severe inattentive symptoms were associated with decreased self-perception in terms of scholastic and athletic competence, both of which are related to school activities and classes. Children with severe inattention are more likely to make mistakes on schoolwork and in physical education classes in school, and to be reproached by teachers and parents (37). These reproaches could decrease self-perception in some domains, while individuals may maintain healthy self-perception for other domains, such physical appearance, that are not as likely to draw rebukes from others.

Hyperactive–impulsive symptoms were associated with self-perception in terms of Behavioral Conduct. This domain was evaluated *via* several questions, such as “I often do something that I know I should not do” and “I think that I cannot behave nicely.” Hyperactive–impulsive children often cannot keep themselves from doing things they should not do. They may regret their behaviors later on, particularly when they get scolded by their teachers and other adults for what they have done [e.g., Ref. (38)]. The combination of remorse and reproaches from others may decrease their self-perception regarding behavioral conduct.

Low self-esteem was associated with more severe depressive symptoms. Many previous findings have indicated that children with ADHD have low self-esteem, which can lead to depression (24, 25). These internalized problems become pronounced during the transition from childhood to adolescence, and some children with ADHD are first diagnosed with depression during adolescence (39, 40). Additionally, it is interesting to note that depression was associated with low self-perception with respect to Social Acceptance and Physical Appearance, which were not influenced by ADHD symptoms. Nishikawa et al. (41) also proposed non-academic self-concept (i.e., social and physical factors) as a predictor of internalizing and externalizing problems in Japanese adolescents. Japanese adolescents assign more importance to friendships and physical appearance compared with school activities such as scholastic and athletic competence. This is likely because Japanese people traditionally emphasize group harmony and compliance with social norms (42, 43). Thus, such culturally imbedded attitudes can have a potent influence on the mental health of Japanese adolescents, for instance, making them more susceptible to depressive symptoms if they have low self-perception regarding social and physical appearance.

Oppositional defiant disorder symptoms were directly associated with depressive symptoms, while ADHD symptoms were indirectly associated with depression *via* self-esteem. Recently, Leadbeater and Homel (44) reported that ODD symptoms among adolescents were linked with both internalizing (anxiety and depression) and externalizing (conduct problems) problems. Boylan et al. (45) also indicated that ODD is often comorbid with internalizing disorders (anxiety/depressive disorder) in childhood and adolescence. The present results are consistent with these previous findings and suggest that ODD symptoms might influence depression directly, unlike the relationship between ADHD symptoms and depression mentioned above. Thus, adolescents with ODD symptoms may be at risk for depression, even though their ODD symptoms have a minimal influence on self-esteem and self-perception.

We found gender differences in ADHD symptoms in our sample of Japanese early adolescents. Specifically, boys received higher Inattention and Hyperactive–Impulsivity ratings than girls, while we found no gender differences in ODD symptoms. ADHD symptoms are usually more pronounced in boys compared with girls, regardless of whether measurements are made using parent-rated or teacher-rated scales (46, 47). These gender differences tend to decrease with age and are not found among adults in countries other than Japan (46, 48). However, the degree of ADHD symptoms in healthy samples can vary from country to country, for example, Japanese university students have higher ratings of inattention than university students in the USA (49). Further studies, based on cultural differences, are needed to confirm whether gender differences in ADHD symptoms decrease from adolescence to adulthood in Japanese samples.

The present study has several limitations. First, ADHD symptoms were evaluated only by teachers and not by parents. Behaviors and mental states of adolescents may vary widely between home to school. Indeed, ADHD symptoms in the present study might have been overestimated or underestimated by teachers, who based their evaluations only on performance at school. Future studies would benefit from examining the gaps between teacher-rated and parent-rated ADHD symptoms and investigate the relationships between parent-rated ADHD symptoms and other factors, such as self-esteem and depression. Second, we did not consider information about the family environment of the adolescents, such as socioeconomic state or parenting attitude. These family factors can have a major impact on mental and physical health in children from infancy to adolescence [e.g., Ref. (50)]. Further studies should include this information to assess the influences both of personal (i.e., ADHD and ODD symptoms) and environmental factors (i.e., family social environment) on self-esteem and depressive symptoms. Third, we did not find any influences of comorbidity of ADHD and ODD on self-esteem, self-perception, and depression. Previous researches indicates high rate of the comorbidity of ADHD and ODD (51, 52), and the present study also found significant correlations between ADHD and ODD symptoms. However, the SEM including the interaction effect of the comorbidity did not produce good-fit indices, meaning that the interaction effect was not confirmed on the present samples. We should examine the influences of comorbidity on self-esteem and depression when we conduct next study on clinical samples. Finally, the present research was a cross-sectional study which does not enable us to confirm causalities among the variables. In the present study, we were aware of the limitations of the cross-sectional study and estimated the associations between the variables based on the statistical analyses. A longitudinal study (i.e., a cohort study) is needed to confirm the causalities that are suggested in the present study.

In conclusion, to the best of our knowledge, this is the first study to examine the relationships among ADHD and ODD symptoms, self-esteem, self-perception, and depressive symptoms in adolescents. Inattentive symptoms were associated with self-esteem and self-perception in terms of scholastic and athletic competence, while hyperactive–impulsive symptoms were associated with self-perception regarding behaviors. However, we did not find a direct effect between ADHD symptoms and depressive symptoms in adolescents. Low self-esteem and self-perception were strongly associated with depression. ODD symptoms, however, were directly associated with depression, without the mediating effects of self-esteem. The present findings indicate that inattentive symptoms have a negative influence on self-esteem and depressive symptoms, even if adolescents have subthreshold ADHD symptoms. Additionally, adolescents with severe ODD symptoms may be at greater risk for depressive symptoms.

## Ethics Statement

This study was carried out in accordance with the recommendations of “Ethical Guidelines for Medical and Health Research Involving Human Subjects, the ethics committee at the Shimada Ryoiku Center Hachioji (Tokyo, Japan; approval number 201503)”; with written informed consent from all subjects. All subjects gave written informed consent in accordance with the Declaration of Helsinki. The protocol was approved by the “the ethics committee at the Shimada Ryoiku Center Hachioji (Tokyo, Japan; approval number 201503).”

## Author Contributions

YK and YI contributed to the design of the study and the data collection, analyzed and interpreted the data, drafted the manuscript, agreed to the final version of the manuscript, and agreed to be accountable to all aspects of this work.

## Conflict of Interest Statement

The authors declare that the research was conducted in the absence of any commercial or financial relationships that could be construed as a potential conflict of interest.
